# IL10-modified Human Mesenchymal Stem Cells inhibit Pancreatic Cancer growth through Angiogenesis Inhibition

**DOI:** 10.7150/jca.38062

**Published:** 2020-07-09

**Authors:** Chunyan Zhao, Yu Pu, Haidi Zhang, Xianhua Hu, Rendan Zhang, Shuai He, Qi Zhao, Bo Mu

**Affiliations:** 1 Sicuhan Key Laboratory of Medical Imaging, Affiliated Hospital of North Sichuan Medical University 637000, Nanchong, Sichuan Province, China.; 2 School of Preclinical Medicine, North Sichuan Medical University 637000, Nanchong, Sichuan Province, China.; 3 Department of Clinical Medicine, North Sichuan Medical University 637000, Nanchong, Sichuan Province, China.

**Keywords:** IL10, hMSC, Angiogenesis, Pancreatic cancer

## Abstract

In the present study, we constructed the recombinant plasmid IL10-PEGFP-C1 and successfully transfected into human mesenchymal stem cells. After culturing for 72 h, the levels of IL6 and TNF-α in the supernatant of the MSCs-IL10 group were significantly lower than the vector group and the control group (17.6 ± 0.68vs73.8 ± 0.8 and 74.4 ± 1.5) µg/L and (65.05 ± 3.8 vs 203.2 ± 2.4 and 201.3 ± 3.7) µg/L, respectively (p < 0.001) .The animal experiments showed that the volume of subcutaneous tumors in the MSCs-IL10 group *in vivo* was a significantly less level compared to that in MSC control and the blank control groups (76.84 ± 20.11) mm^3^ vs (518. 344 ± 48.66) mm^3^, (576.99± 49.88) mm^3^, (P < 0. 05) and they have a longer life time. Further we found the mass concentrations of IL6 and TNF-α in the blood serum of MSC-IL10 group were lower than the vector group and the control group (64.42 ± 10.9 vs120.83 ± 15.52 and 122.65 ± 13.71) and (40.05 ± 5.63 vs 126.78 ±1.89 and 105.83 ± 2.16) µg/L respectively (p < 0.001). CD31 immunohistochemistry and alginate encapsulation experiments showed tumor angiogenesis were inhibited in MSCs-IL10 group in comparison to the control and vector group (P < 0.001), FITC-labeled dextran intake was also lower than the other groups (P < 0.01). Collectively, this study suggested IL10 could inhibit the growth of the transplanted tumor *in vivo* and prolong survival of mice, and the primary mechanism may be the indirect inhibition of pro-inflammatory cytokines IL6 and TNF-α secretion and tumor angiogenesis formation.

## Introduction

In the Western world, the fourth highest reason of deaths due to cancer is pancreatic cancer and it is predicted to be the second most common reason around 2030 1. Although prior research has shown that several cancers in humans are caused directly due to chronic inflammation and also contribute to angiogenesis 2, their mechanisms are not fully elucidated.

Folkman et al. hypothesized the dependence of tumor growth on angiogenesis 3, proven subsequently and deemed an important milestone in cancer research 4. As a result, antiangiogenic regimens were shown to be potent against multiple solid tumors, including clear cancers of the renal cell 5, the ovary 6, and the cervix 7. The characteristics of PDAC (Pancreatic Ductal Adenocarcinoma) include extremely high potential for metastasis and invasion, besides 8 angiogenesis which has a vital role in this process. IL-10 (Interleukin-10) is apparently a cytokine that is potentially anti- inflammatory. It is produced by almost all the cells of innate and adaptive immunity, which also act as its targets, implicating the regulation and compartmentalization of IL-10 secretion and its activity. However, the half-life of IL-10 in the body is very short. How to transport IL10 to the tumor microenvironment is an urgent problem to be solved. MSCs (mesenchymal stem cells) that have been genetically modified are hypothesized to show potential therapeutic abilities in several human diseases, including cancer. MSCs expressing IFN -β (interferon-β) injected intravenously may impede the pulmonary metastasis expansion of breast cancer and melanoma in mice 9-11, and increase the survival of mice having glioma xenografts 12. The intrinsic homing ability is a well-accepted and a vital property of MSCs in cellular therapies. When systemically infused, MSCs are able to establish on the sites of injury, tumor, ischemia, and inflammation, while the inherent molecular mechanisms remain to be unraveled 13, 14.

In the current study, we evaluate the potential of using genetically modified MSCs which express IL10 constitutively to impede the pancreatic cancer cells proliferation *in vitro* and reduce the growth of tumor xenograft *in vivo*. We also examined the mechanisms of action; our approach likely targets pancreatic cancer microenvironment, and inhibits angiogenesis.

## Materials and Methods

### Animals and Cell Lines

The Experimental Animal Center (West China) supplied us with BALB/c mice (6 to 8 week old). Approved consent for these animal experiments was given by the Medical Ethics Examination Approval of North Sichuan Medical University.

The cell-line human PANC-1 was procured from ATCC (American Type Culture Collection) and further grown in DMEM containing fetal bovine serum (FBS, 10%) (both from Invitrogen, USA), L-glutamine (2 mM), and antibiotics penicillin (100 units/mL), and streptomycin (100 μg/mL) at 37 °C in an incubator adjusted with 5% CO2.

The procurement of human BMSC was done from Saiqi Biological Engineering Co., Ltd. (Shanghai) and grown as per instructions of the manufacturer in L-DMEM from Gibco (Grand Island, USA) and 10% FBS.

The hIL10 over-expression construct plasmid in presence of Lipofectamine 2000 reagent from Invitrogen (USA) was transfected in BMSCs as per instructions of the manufacturer.

### qRT-PCR assay

The cultured cells from each group were used to isolate total RNA using TRIzol from TAKARA Biotechnology, (Dalian, China). The expression of IL10 gene was measured using a real-time PCR system, ABI Prism 7300 from Applied Biosystems (USA) and SYBR Green from TAKARA Biotechnology, the IL 10 gene expression was measured. Specific primers were used to amplify each target gene from the cDNA. The program for real-time qPCR (Quantitative reverse transcription polymerase chain reaction) was: 95 °C for 10 min, 35 cycles for 15 s at 95 °C, and for 31 s at 60 °C, and then a dissolution curve was included. The Primer 5.0 software (Applied Biosystems) was used to design primers and their respective sequences are: IFN-α2b forward, 5′-TCCAAAAGGCTGAAACCATCC-3′ and reverse, 5′-GACAACCTCCCAGGCACAAG-3'. To control the variations in the expression levels the data for expression were normalized with the housekeeping gene β-actin geometric mean, amplified using the primers: forward, 5′ACGGCAAGTTCAACGGCACAG-3′ and reverse, 5′-GACGCCAGTAGACTCCACGACA-3′. The comparison of efficiency of amplification between the reference and the target was done using the ΔΔCt calculation.

### Western blot assay

Cell lysis was performed using the buffer from Kangwei Biotechnology (China). Proteins, in equal amounts were then resolved by SDS-PAGE, and transferred to a nitrocellulose membrane supplied by Whatman (Germany). Further incubation of membranes at 4 °C in the presence of primary antibody (Santa Cruz Biotechnology) was done overnight, followed by incubation at room temperature with secondary antibody for 2 h. For all the experiments, the loading control was ß-actin (1:2000) from Santa Cruz Biotechnology (CA, USA). The bands were then observed using an enhanced chemiluminescent reagent manufactured by Beyotime (Beijing, China).

### Enzyme-linked Immunosorbent (ELISA) Assays

ELISA was done to assess the TNF-α and IL-6 levels in the mice sera and cellular culture as per instructions of manufacturer's (R&D Systems, Inc., Shanghai, China).

### Immunohistochemistry (IHC)

The overnight incubation of sections with primary CD31 antibody at 4 °C was done and were then incubated at room temperature with secondary antibody (1:500) conjugated with HRP (horseradish peroxidase) for 2 h. The visualization of samples incubated with secondary antibody (HRP-conjugated) was done by developing in DAB reagent from Boster Biological Technology (China), followed by counterstaining of the nuclei using hematoxylin. Observation of sections was done under a light microscope from Olympus (Japan); the brown stain indicated a positive antibody expression was indicated by brown stain, and the nuclei were stained in blue.

### Assay using alginate-encapsulated tumor cells

This assay was done to assess the role of MSC-IL10 in impeding tumor cells-induced angiogenesis *in vivo*, as previously described 15. In brief, 5×10^4^ PANC-1 cells were encapsulated in each alginate bead and subcutaneously implanted in athymic mice into both dorsal flanks. From the day 2, the mice were treated with MSC-IL10 (5×10^5^ cells), MSC-vector, MSC and PBS. After 14 days of implantation, injection of fluorescence isothiocyanate (FITC)-dextran solution from Sigma (MO, USA) was done into the tail vein of the mice at 100 mg/kg dosage. After 20 min of injection, alginate beads were extracted and visualized.

### Animal studies

The Institutional Animal Care and Treatment Committee, North Sichuan Medical College, China consented to all animal experiments. The tumors of PANC-1 were established by injecting 5×10^6^ cells (100 μL) into the dorsal area of athymic female nude mice (balb/c, nu/nu; 6 to 8-week-old). The mice were randomized (6/group) when tumors attained about 100 mm^3^ volume, and once daily, MSC-IL10, MSC-vector, MSC and PBS were injected in tail vein of mice. Every three days, growth of tumor was evaluated in the period of treatment. Volumes of tumors were calculated as per the formula: volume (mm^3^) = width (mm^2^) ×0.5 × length (mm). From the start of treatment, inhibition of growth was determined by comparing the mean change in tumor volume in the treated and vehicle groups.

### Survival analysis

To further understand the effect of IL10 gene-modified MSCs on the survival of PANC-1 tumor-bearing mice, 30 PANC-1 subcutaneous tumor models were randomly categorized into MSC, MSC-vector and MSC-IL10 groups, with 10 mice in each group. The treatment plan was completely related to the efficacy of subcutaneous tumors. The time of death of each mouse was recorded.

### Statistical analysis

All values are presented as mean ±SD (standard deviation). Significant differences in multiple comparisons were identified by one-way ANOVA (analysis of variance), along with Student's *t* test (one-tailed). A statistically significant value fulfilled the criterion of *P* <0.05.

## Results

### High-level of IL10 expression is induced by BMSC/IL10 cells

Although in some diseases therapy and research, MSCs modified by IL10 gene have been applied 16, in-depth study using BMSCs modified by IL10 have not been carried out about cancer. For this, we first assessed the IL 10 transfection efficiency in BMSC.

**Figure 1:**
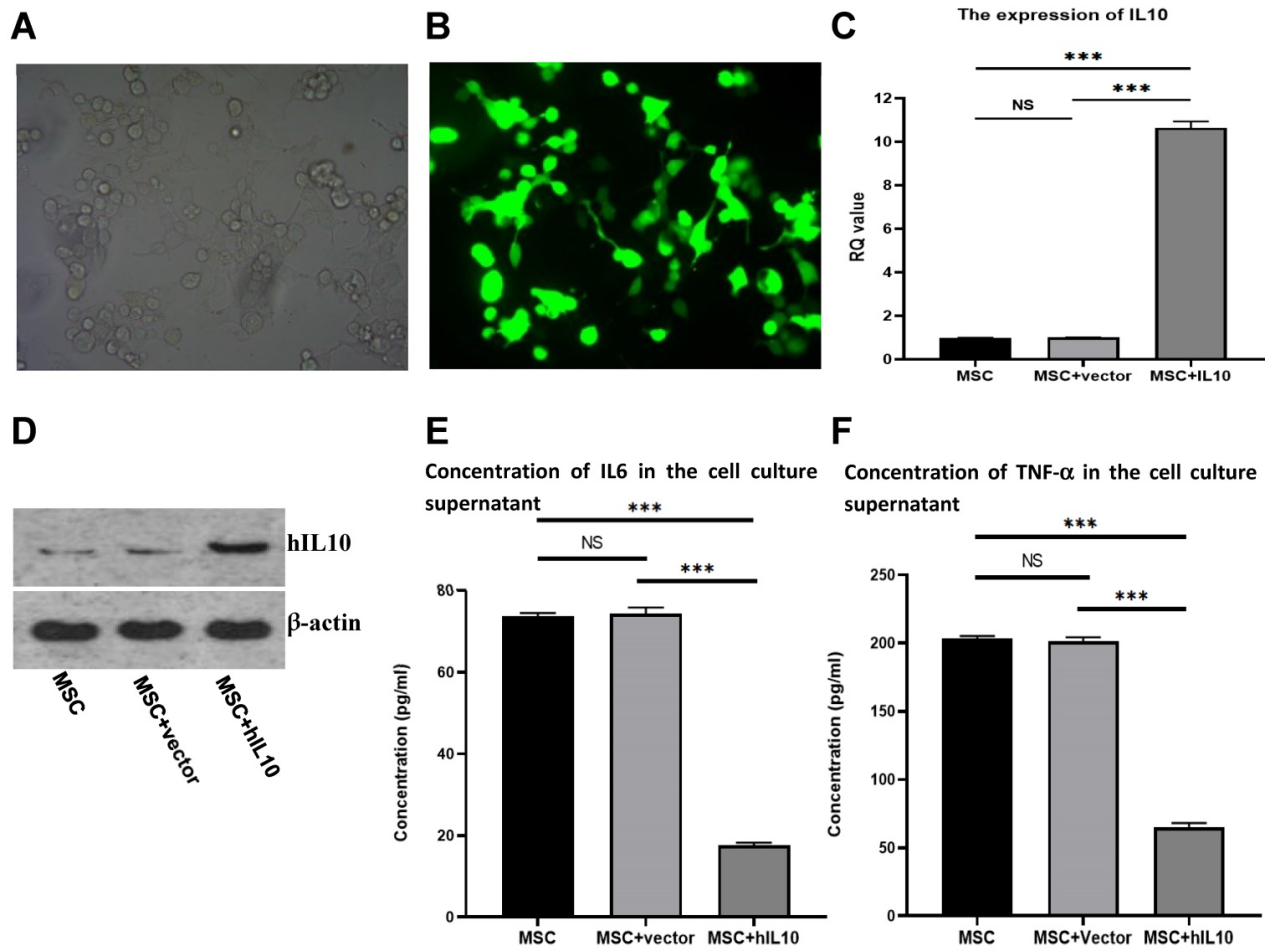
Stable expression of IL10 gene in BMSC following transfection with IL10-bering plasmid and inhibitory effect of MSC-IL10 on inflammatory cytokines in MSC. MSC modified with IL10 gene photographed under light microscope (×200). MSC modified with IL10 gene photographed under fluorescent microscope (×200). RT-PCR analysis for IL10 from MSC-IL10 as well as MSC and MSC-vector transfected groups. Western blot analysis for IL10 from MSC-IL10 as well as MSC and MSC-vector transfected groups. A significant decrease in IL6 was observed in the supernatant of MSC-IL10 group in comparison to the other groups (***P<0.001). A significant decrease in TNF-α was observed in the supernatant of MSC-IL10 group in comparison to the other groups (***P<0.001).

IL10/pEGFP-C1 plasmids were transfected in BMSC. The field of view under the fluoroscopy was compared with the field of view under the light microscope (the number of cells under white light and fluorescence were counted separately, and compared). After 72 h, the transfection efficiency of the cells was over 80% (Figure 1A and 1B), and IL10 mRNA expression in BMSC enhanced nearly 10-fold in comparison to control, as assessed by q-PCR (Figure 1C). Moreover, IL10 concentration in the medium increased nearly four-fold compared to the control group (Figure 1D), as assessed by western blot analysis.

### Effect of IL10 gene on the expression of cytokines in human MSCs

The adult stem-cells, also known as MSCs secrete various cytokines 4. To evaluate the effect of the cytokine secretion of human mesenchymal stem cells with modification of IL10 gene, we determined the secretion capacity of cell inflammatory cytokines through ELISA. As shown in Figure 1, the concentrations of IL6 (Figure 1E) and TNF-α (Figure 1F) in the supernatant MSC-IL10 group were 17.6 ± 0.68 µg/L and 65.05 ± 3.8 µg/L, respectively after 72 h. The concentrations of IL6 and TNF-α in the supernatant of MSC-vector and blank control groups were 73.8 ± 0.8 µg/L, 203.2 ± 2.4 µg/L and 74.4 ± 1.5 µg/L, 201.3 ± 3.7 µg/L respectively. Therefore, over expression of IL10 gene significantly reduced the expression of IL6 and TNF-α in MSC.

### MSC-IL10 prolongs survival in mice with tumors by inhibiting subcutaneous tumor growth

**Figure 2:**
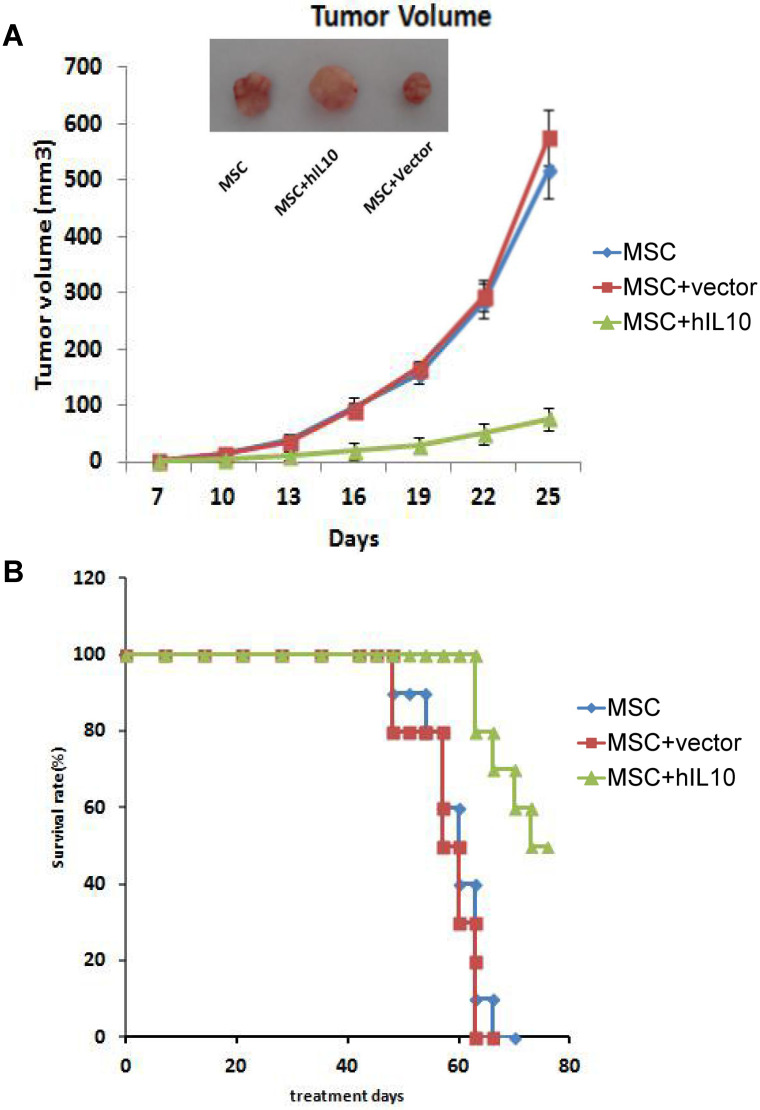
The role of MSC-IL10 on tumor growth inhibition in xenograft Balb/c Mouse models. A significant variation (P<0.05) in volume of tumors was observed between MSC-IL10 treated and the other mice groups. The tumor growth in terms of mean ± SEM of five mice is depicted. A significant enhancement in survival was observed in MSC-IL10 treated mice, in comparison to the other treated mice groups (P<0.05).

To assess the role of MSCs modified by IL10 on mice with tumors *in vivo*, we established a subcutaneous mice model with pancreatic cancer, MSC-IL10 was injected into the tail vein once and the effect on tumor growth on mice was evaluated. As shown in Figure 2, MSC-IL10 obviously inhibited tumor growth compared with other groups. Meanwhile, the period of survival was prolonged for PANC-1 mice with tumors.

### MSC-IL10 reduced the TNF-α and IL-6 expressions in the blood of mice with tumors

**Figure 3:**
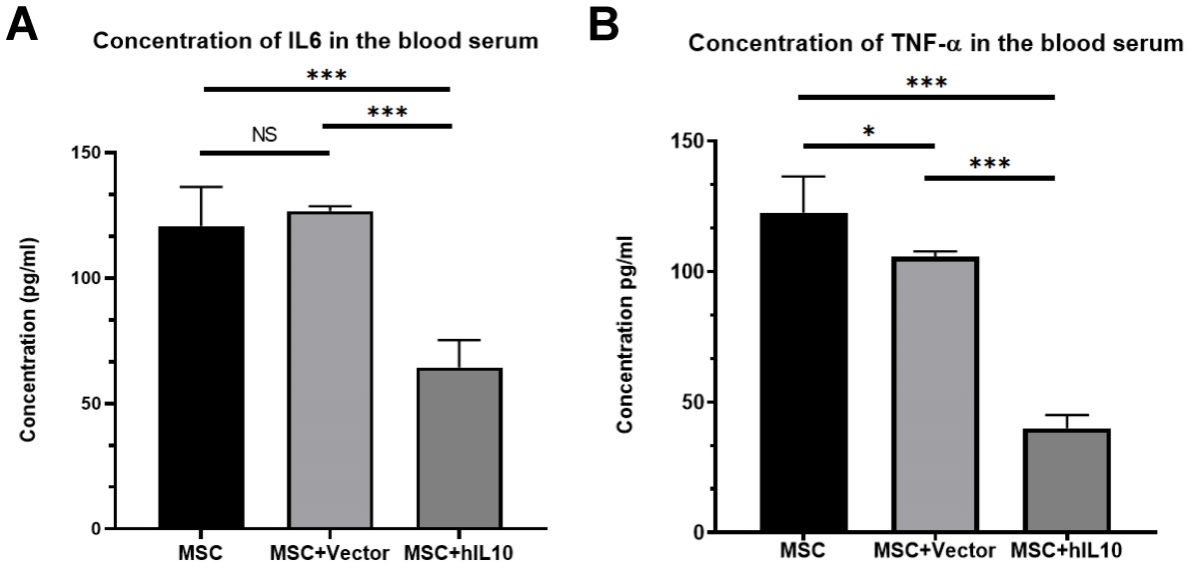
The inhibitory effect of MSC-IL10 on inflammation cytokines in blood of mice. IL6 was a significant decrease in the serum of MSC-IL10 group mice in comparison to the other mice group (***P<0.001). TNF-α was a significant decrease in the serum of MSC-IL10 group mice in comparison to the other mice group (***P<0.001).

We performed ELISA assay to determine the expression of IL6 and TNF-α in the blood of tumor-bearing mice. Results show the mass concentrations of IL6 and TNF-α in serum were 64.42±10.9 µg/L and 40.05±5.63 µg/L, respectively (Figure 3). Those in MSC, MSC-vector group were 120.83±15.52 µg/L, 126.78±1.89 µg/L and 122.65±13.71 µg/L and 105.83±2.16 µg/L, respectively. Therefore, over expression of IL10 gene significantly reduced the expression of inflammation cytokines in the blood of the mice with tumors (P < 0.001).

### MSC-IL10 inhibited tumor angiogenesis

**Figure 4:**
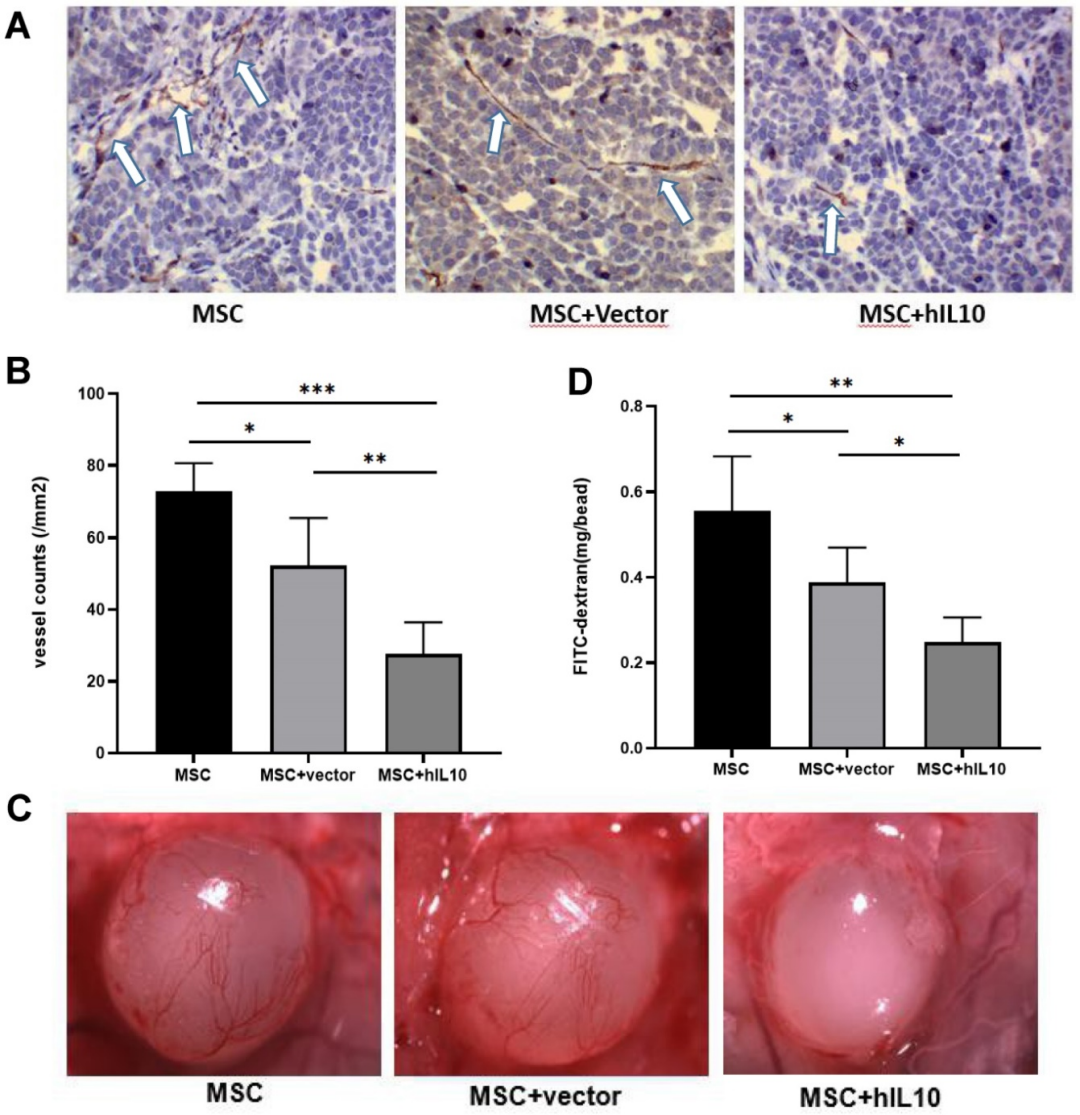
MSC-IL10 mediated inhibition of angiogenesis *in vivo*. (**A and B**) Immunohistochemical analysis with the anti-CD31 antibody to show significant inhibition of tumor microvessels by MSC-IL10 in PANC-1 tumor xenografts. Vasculature of tumors from vehicle and MSC-IL10 treated mice are presented. Vascular structure indicated by white arrows. For each tumor, the average of fields were calculated and the averages for each animal were used to determine the final mean ±SD (n = 6 mice/group); (*P <0.05, **P<0.01, ***P<0.001). (**C and D**) Quantification of significant inhibition of angiogenesis in mice transfected with MSC-IL10 in alginate beads containing PANC-1 tumor cells and FITC-dextran. The beads from MSC-IL10-treated mice showed a significant decrease in FITC-dextran uptake in comparison to the vehicle group. Data are presented as mean ± SD (n = 6 mice/group); (*P <0.05, **P<0.01).

Furthermore, the mechanism of *in vivo* growth inhibition of pancreatic cancer cells by MSC modified by IL10 was studied using immunohistochemistry and alginate assays, which confirmed that MSC-IL10 inhibited the formation of angiogenesis in tumors (Figure 4).

The results showed tumor angiogenesis were inhibited in mice with MSCs-IL10 compared to the mice injected with empty-vector, as well as only MSC (P < 0.001), and the FITC-labeled dextran intake was also lower than that in the other groups (P < 0.01).

## Discussion

Tumor progression is dependent on the production of a new vascular network to supply its nutrients. As a disease dependent on vascular system, angiogenesis in tumors is crucial in the progression, development, and metastasis of pancreatic cancer 17-19. If angiogenesis of pancreatic cancer is effectively prevented, it may potentially control tumor recurrence and metastasis after surgery or radiotherapy and chemotherapy 20-22.

Because tumor endothelial cells are genetically stable and resistant to drug, efficient anti-tumor angiogenesis therapy has become one of the promising treatments in the field of cancer therapy. A variety of drugs that inhibit neovascularization have been used for anti-tumor research 23-25.

IL10 has potent anti-angiogenic activity and can inhibit the growth and metastasis of tumor cell by promoting apoptosis and inducing tumor cell differentiation 26. The above physiological characteristics laid the foundation for the application of IL10 in tumor therapy.

At present, several experimental studies report promising results of IL10 which can significantly inhibit tumor growth and metastasis in a variety of tumor models including pancreatic cancer 26-28. However, viral or non-viral vector gene therapy, due to the lack of targeting, is difficult to penetrate the vascular wall and penetrate into tumor tissue. The purified recombinant protein is easily degraded in the body, requiring long-term and repeated administration; meanwhile it is difficult and expensive to prepare and purify the protein. These shortcomings greatly reduce their potential therapeutic effects and application value of IL10 protein 29-31.

Therefore, effective transportation of IL10 gene to the tumor site and its continuous local expression is the focus of this study. Since last few years, MSCs are extensively used in gene therapy for tumors as an ideal cell vector because of the low immunogenicity, ability of homing to tumor tissues, and easy isolation, culture, expansion and genetic modification of MSCs 32.

Previous studies on some disease models have shown that IL10-modified mesenchymal stem cells exhibit value in various aspects, such as induction of autophagy and neuroprotection. However, previous cancer in-depth studies have not been carried out using BMSCs modified by IL10. For this, we first assessed possibility of IL10 modified mesenchymal stem cells for pancreatic cancer. In our studies, MSCs-IL10 exhibited a strong inhibitory effect on the growth of PANC-1 subcutaneous tumors, and can well inhibited secretion of TNF-α and IL6, the pro-inflammatory cytokines and tumor angiogenesis, so that tumor-bearing mice had longer survival. Therefore, MSCs are a relatively perfect transport vector that protects the IL10 gene from being eliminated in the circulatory system and concurrently not perturbing other tissues because of its high concentration in the blood. Accordingly, the therapeutic effect of IL10 is also promoted. These results indicate that the use of MSCs as a gene vector is a promising tumor treatment strategy.

In summary, the study shows that IL10 can inhibit the growth of transplanted tumor *in vivo* and the main mechanism may be the IL10-medited indirect inhibition of pro- inflammatory cytokines IL6 and TNF-α secretion and tumor angiogenesis. This study also shows that it is feasible to transduce IL10 gene into MSC, which lays a foundation for the clinical application of IL10 to anti-angiogenic gene therapy in pancreatic cancer.
